# Co-existing of craniofacial fibrous dysplasia and cerebrovascular diseases: a series of 22 cases and review of the literature

**DOI:** 10.1186/s13023-021-02102-x

**Published:** 2021-11-04

**Authors:** Xiaowen Song, Zhi Li

**Affiliations:** 1 Department of Neurosurgery, Beijing Tiantan Hospital, Capital Medical University, No 119, Nansihuan xilu, Beijing, 100070 China; 2 China National Research Center for Neurological Disease, Beijing, China

**Keywords:** Fibrous dysplasia, Cerebrovascular diseases, Coexistence, Interaction

## Abstract

**Background:**

Craniofacial fibrous dysplasia is a fairly rare condition. Some literature have reported a few patients with craniofacial fibrous dysplasia suffering from vascular abnormalities. This study aimed to describe the possible coexistence of craniofacial fibrous dysplasia and cerebrovascular diseases for the first time.

**Method:**

We retrospectively reviewed the 1175 patients with craniofacial fibrous dysplasia in Beijing Tiantan Hospital and the information of the 22 patients coexisted with cerebrovascular diseases were described. In addition, we performed a systematic review for cases of craniofacial fibrous dysplasia with vascular abnormalities.

**Result:**

22 out of 1175 patients (1.9%) were diagnosed with craniofacial fibrous dysplasia and cerebrovascular diseases including 9 intracranial aneurysms, 4 venous malformations, 2 arteriovenous malformations, 1 moyamoya disease, 2 intracranial venous stenosis and 4 cerebral ischemia with a mean age of 38.18 years old. Only 2 patients were managed surgically for craniofacial fibrous dysplasia and 6 patients were treated with neurosurgery for cerebrovascular diseases. 8 patients were closely followed and only 1 patient’s symptoms worsened.

**Conclusion:**

Craniofacial fibrous dysplasia might cause constriction of the intracranial vessels and alteration of the overall hemodynamics of the intracranial vasculature resulting in various cerebrovascular diseases. Multimodal screening and examinations seems reasonable for patients with craniofacial fibrous dysplasia for throughout treatment and prognosis evaluations.

## Background

Fibrous dysplasia (FD) is a non-malignant condition in which normal bone and marrow are replaced by fibrous tissue and haphazardly distributed woven bone [1]. Patients may exhibit involvement of one bone (monostotic FD; MFD), multiple bones (polyostotic FD; PFD) or they may have McCune-Albright syndrome (MAS). The most common locations affected are the craniofacial bones, proximal femur, and rib [2], with the craniofacial region involved in 90% of the PED and MAS cases and the anterior cranial base involved in over 95% of these cases [3]. Depending on the type and location of FD, the signs and symptoms vary and include facial deformity and asymmetry, vision changes, hearing impairment, nasal congestion and/or obstruction, pain, paresthesia, and malocclusion.

Vascular abnormalities complicating FD has been reported in several case reports [4–10], suggesting that although there was no known common etiology, these two kinds of entities might be related to some extent. However, there was no literature focusing on the co-existence of craniofacial fibrous dysplasia (CFD) and cerebrovascular diseases (CVD), considering that the number of patients with CFD is pretty limited. Here we present a series of patients diagnosed with CFD coexisting with CVD which has been rare and never been reported before and the possible interactions and relationships between these two diseases were deduced, analyzed and discussed. And a literature review was done to further invest the coexistence of CFD and vascular diseases.

## Methods

We retrospectively searched the radiological database in Beijing Tiantan Hospital and 1175 patients were found diagnosed with CFD radiologically during 2003–2020. And we further reviewed the medical records and radiological examinations of these 1175 patients. 22 patients coexisted with CVD were identified and were divided into the following two groups: hemorrhagic cerebrovascular diseases including intracranial aneurysm (IA) and vascular malformation and ischemic cerebrovascular diseases including vascular stenosis/occlusion and cerebral ischemia. All the CFD patients were diagnosed with classical radiological characteristics. And the clinical and radiological characteristics of these 22 patients were collected and described, and the possible relationships between the two diseases were discussed.

In addition, we searched 3 medical database, PubMed, EMBASE and Cochrane Library up to 2020 for published studies focusing on the coexistence of CFD and vascular diseases. The following combined terms ([MESH] “fibrous dysplasia” AND [MESH] “cerebrovascular diseases” or “intracranial aneurysm” or “cerebrovascular malformation” or “moyamoya disease” or “cerebral ischemia” or “intracranial hemorrhage”). A manual researching on the reference of identified studies was performed for more related studies.

This study was approved by Institutional Review Board of Beijing Tiantan Hospital, Capital Medical University. Due to the retrospective nature of our study, the board waived the need for written consent.

## Results

### Clinical characteristics

Of the 1175 FD patients evaluated, there were 22 patients (1.9%) coexisted with CVD, with 15 falling into the hemorrhage group among whom 9 were diagnosed with IAs, 6 with cerebrovascular malformations (4 venous malformations and 2 arteriovenous malformations (AVMs)) and the other 7 divided into the ischemia group among whom 1 was diagnosed with moyamoya disease, 2 with intracranial venous sinus stenosis and 4 with cerebral ischemia. The mean age of two groups of patients was 37.27 (ranging from 6 to 65) and 40.14 years old (ranging from 25 to 56) respectively, with a mean age of 38.38 years old for all the included patients. Only 2 (0.9%) were pediatric patients (aged ≤ 18 years). The gender ratio was female: male = 1.9:1. Only 2 out of the 22 patients were treated with transnasal surgery for FD and 6 patients were managed surgically for cerebrovascular diseases. All of the patients were discharged home in good condition no matter what treatment was done. Chest-X ray and Echocardiography were screened for all the included patients and 2 patients were found to have anomalies of ribs indicating PFD. None of them had endocrinopathies or was diagnosed with MAS or other known FD related diseases such as breast cancer and intraductal papillary mucinous neoplasms (IPMNs). The detailed clinical information was presented in Table 1 and Table 2.

**Table 1:** Information of patients with hemorrhagic cerebrovascular diseases

Patient NO	Gender	Age	Cerebrovascular disease		Fibrous dysplasia	Neurovascular impingement		Management		Clinial symptoms	Multisystem involvement	Follow-up	Prognosis	
			Type	Localization		Vasculature	Nerve	CVD	CFD				CVD	CFD
Case 1	male	39	IA	LICA,C6	Right sphenoid bone, clival and sellar region	Displacement of RICA	bilateral optic canals	Follow-up and observation	Transnasal surgical resection	Temporal hemianopsia	No	No		
Case 2	female	26	IA	BICA,C7	Right temporal bone, sellar region, orbit and pterygoid process	neighboring to RICA	no	Follow-up and observation	Follow-up and observation	asymptomatic	No	No		
Case 3	female	34	IA	RICA, C6	Left sphenoid bone	neighboring to LICA	Left optic canal	Follow-up and observation	Follow-up and observation	headache	No	No		
Case 4	male	53	IA	RICA, C6	Sphenoid sinus, sella and left orbital apex	Displacement of LICA	Left optic canal	Aneurysm embolization	Follow-up and observation	headache	No	6 years	No recurrence	unchanged
Case 5	female	62	IA	BMCA	Left temporal bone and sphenoid bone	neighboring to LICA	No	Follow-up and observation	Follow-up and observation	headache	No	No		
Case 6	female	64	IA	LICA,C7	Right temporal bone	neighboring to RICA	No	Aneurysm clipping	Follow-up and observation	headache	No	1 year	No recurrence	unchanged
Case 7	female	6	IA	BICA, BA	Right frontal,temporal and parietal bones			Follow-up and observation	Follow-up and observation	swelling of left fronto-temporal bone	No	No		
Case 8	female	33	IA	RICA,C5	Left sphenoid bone	neighboring to RICA	No	Follow-up and observation	Follow-up and observation	headache	No	No		
Case 9	female	53	IA	LICA,C7	Left frontal base, ethmoid sinus and left orbit	neighboring to LICA	No	Aneurysm clipping	Follow-up and observation	asymptomatic	No	No		
Case 10	male	65	Venous malformation	Right frontal lobe	Occipital bone and sphenoid bone	neighboring to RICA	No	Follow-up and observation	Follow-up and observation	asymptomatic	No	No		
Case 11	female	13	Venous malformation	Left frontal lobe	Right occipital bone and parietal bone	No	No	Follow-up and observation	Follow-up and observation	Swelling of right parietal bone	No	No		
Case 12	male	20	Venous malformation	Left frontal lobe	Sphenoid bone and left orbital apex	neighboring to LICA	Left optic canal	Follow-up and observation	Transnasal surgical resection	Double vision, vision loss and exotropia of left eye	No	2 years	unchanged	relieved
Case 13	female	30	Venous malformation	Left frontal lobe	Frontal bone, nasal bone,right orbit and right great wing of sphenoid bone	No	No	Follow-up and observation	Follow-up and observation	Swelling of left craniofacial bones	Arachnoid cyst involving right temporal pole	No		
Case 14	male	30	AVM	Right parietal lobe	Sphenoid bone	Encircling of LICA (C4)	No	Craniotomy surgical resection	Follow-up and observation	Vision loss of left eye	Rib FD	1 year	No recurrence	unchanged
Case 15	male	31	AVM	Right fronto-parietal lobe	Right maxillary sinus and right orbit	No	No	Craniotomy surgical resection	Follow-up and observation	Symptoms related with AVM rupture	No	1 year	No recurrence	unchanged

**Table 2:** Information of patients with ischemic cerebrovascular diseases

Patient NO	gender	age	Cerebrovascular disease		Fibrous dysplasia	Neurovascular impingement		management			Clinical symptoms		Multisystem involvement		Follow-up		prognosis		
			type	Localization		vasculature	nerve	CVD	CFD								CVD		CFD
Case 1	Female	34	Moyamoya disease		Right sphenoid bone	neighboring to RICA	No	Indirect revascularization	Follow-up and observation		Recurrent Transient ischemic attack		No		3 years		relieved		Unchanged
Case 2	Female	39	Venous sinus stenosis	Right superior sagittal sinus and right sigmoid sinus	Right sphenoid sinus, right ethmoid sinus and right orbit	Displacement of RICA	Right optic canal	Follow-up and observation	Follow-up and observation		headache		No		No				
Case 3	Male	25	Venous sinus stenosis	Left transverse sinus, sagittal sinus and internal jugular vein	Left ethmoid bone, nasal bone, sphenoid bone, bilateral orbits and frontal bones	Encircling of LICA	Left optic canal	Follow-up and observation	Follow-up and observation		Vision loss of the left eye and weakness of the right limbs		Rib FD		No				
Case 4	Male	44	Cerebral ischemia	Right fronto-temporal lobe, corona radiata and basal ganglia	Right sphenoid bone, frontal–temporal bone and parietal-occipital bone	Encircling of RICA	No	Medical treatment	Follow-up and observation		Swelling of right frontal bones and weakness of left limbs		No		9 years		Progress		Unchanged
Case 5	male	36	Cerebral ischemia	Left basal ganglia and corona radiata	Left parietal bone and temporal bone	Displacement of LICA	No	Medical treatment	Follow-up and observation		Right facial paralysis and aphasia		Diffuse thyroid disease and thyroid nodules		No				
Case 6	male	56	Cerebral ischemia	Right fronto-temporal-parietal lobe	Left frontal bone, orbit and ethmoid bone	Encircling of LICA	Left optic canal	Medical treatment	Follow-up and observation		Numbness of right hand		No		6 months		unchanged		unchanged
Case 7	male	47	Cerebral ischemia	Right fronto-temporal lobe	Sphenoid bone	neighboring to RICA	No	Follow-up and observation	Follow-up and observation		headache		No		No				

### Radiological characteristics

As shown in Table 1 and Table 2, sphenoid bone was the primary site effected by CFD (15/22, 68.2%). Most of the patients presented with slow-growing, painless masses resulting in facial or cranial asymmetry (4/22, 18.2%), and 15.79% (3/22, 13.6%) have vision impairment related with FD of the sphenoid bones.

The position relationships of these two diseases were divided into 4 types: (1) neighboring; (2) displacement; (3) encircling; (4) unrelated. However, of the 9 patients with aneurysms, 6 showed impingement of contralateral internal carotid artery (ICA) instead of the ipsilateral ICA. By contrary, the radiological examinations of all of the 5 patients with cerebral ischemia showed impingement of ipsilateral ICA.

Eight patients were closely followed and most of the CFD stayed unchanged.

### Literature review

Only four studies met the inclusion criteria, all of which were case reports. Two of them were arteriovenous fistulas (AVFs), one was AVM and the other one was middle meningeal artery aneurysm. Detailed information of these four articles were described in Table 3. Although these vascular anormalies seems to be closely related with the corresponding bone lesions, none of them was able to put forward a hypothesis to explain this kind of coexistence.

**Table 3:** Literature review

Author, year	Patient characteristics							Comorbidity	Follow-up
	Age	Gender	Symptom	FD	Vascular disease	Management			
						FD	Vascular disease		
Gianluca [4], 1996	36	Female	Facial asymmetry	MAS; right sphenoid and frontal bones and right ethmoidal cells	Arteriovenous malformation involving the right palpebra and orbit arising from external carotid artery	Follow-up and observation	Follow-up and observation	Dysgenic iridocorneal angles with increased intraocular pressure and retinal degeneration	No
Ishiguro [9], 1985	33	Male	Hard mass	Right frontal bone, orbit, sphenoid bone and parietal bone	Arteriovenous fistula arising from multiple branches of external carotid artery and medial frontal artery	Resection and the defects replaced by bone cement	embolization	No	1 year; no symptoms recur
Yamashita[44] fcv, 2019	66	Female	Cranial bone deformity and vision loss in the left eye	Left cranial vault and left orbit	Middle meningeal artery aneurysm	Follow-up and observation	Surgical removal after Embolization	No	6-month; no symptomatic recurrence
Pan [7], 2020	48	Male	Diplopia, tinnitus	Right sphenoid, occiput, and right temporal bone	Arteriovenous fistula within the occipital FD with blood supply from branches of external carotid arteries and vertebral arteries	Biopsy	Embolization	FD of the right lower extremity, bilateral upper extremeties, ribs, right scapula	No

### Illustrative cases

#### Case 1

**Fig. 1:**
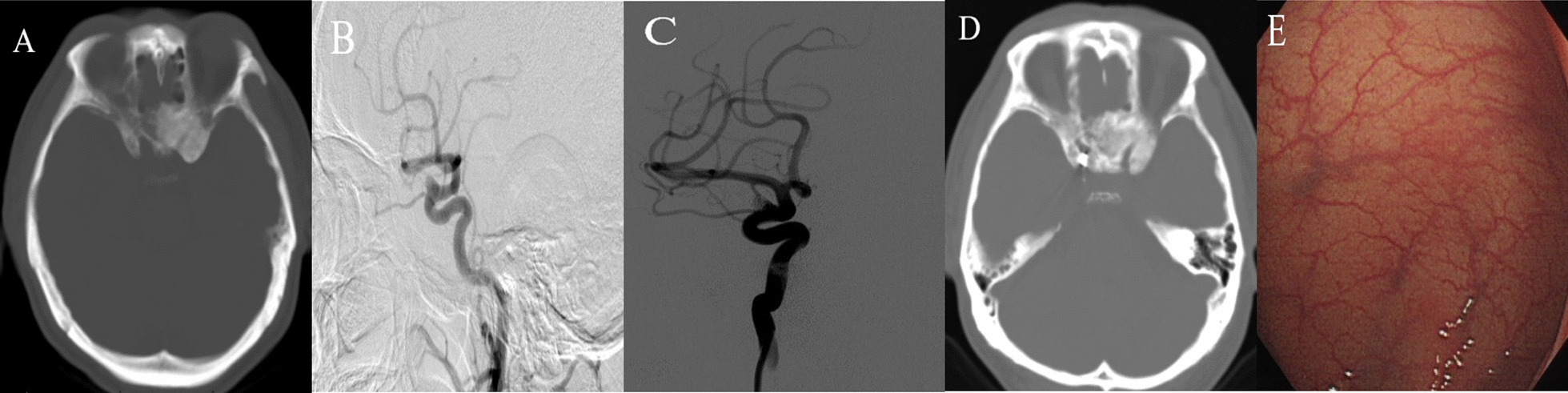
CT showing FD of the left sphenoid bone (**A**) and DSA showing an aneurysm in the right internal carotid artery (C6) (**B**). Stent assisted coil embolization was done and there was no filling of aneurysm (**C**). After 6 years, his FD showed no sign of progression (**D**). At the same time, the patient was diagnosed with colonic vascular malformation by colonoscopy (**E**)

Patient is a 53-year-old male who was admitted into hospital because of interval headache. His was diagnosed with IA for his magnetic resonance angiography (MRA), and Digital subtraction angiography (DSA) was done for a definite diagnosis showing right internal artery aneurysm measuring 7.14 mm*5.63 mm (Fig. 1). At the same time, his magnetic resonance imaging (MRI) and computed tomography (CT) described the abnormal signals in his left sphenoid bone as FD and his colonoscopy found colonic vascular malformations. It was decided to manage the aneurysm with endovascular stent assisted coil embolization. 3 days after operation, the patient was discharged home in good condition. The patient was followed closely for six years and there was no sign of aneurysm recurrence or FD progress.

#### Case 2

**Fig. 2:**
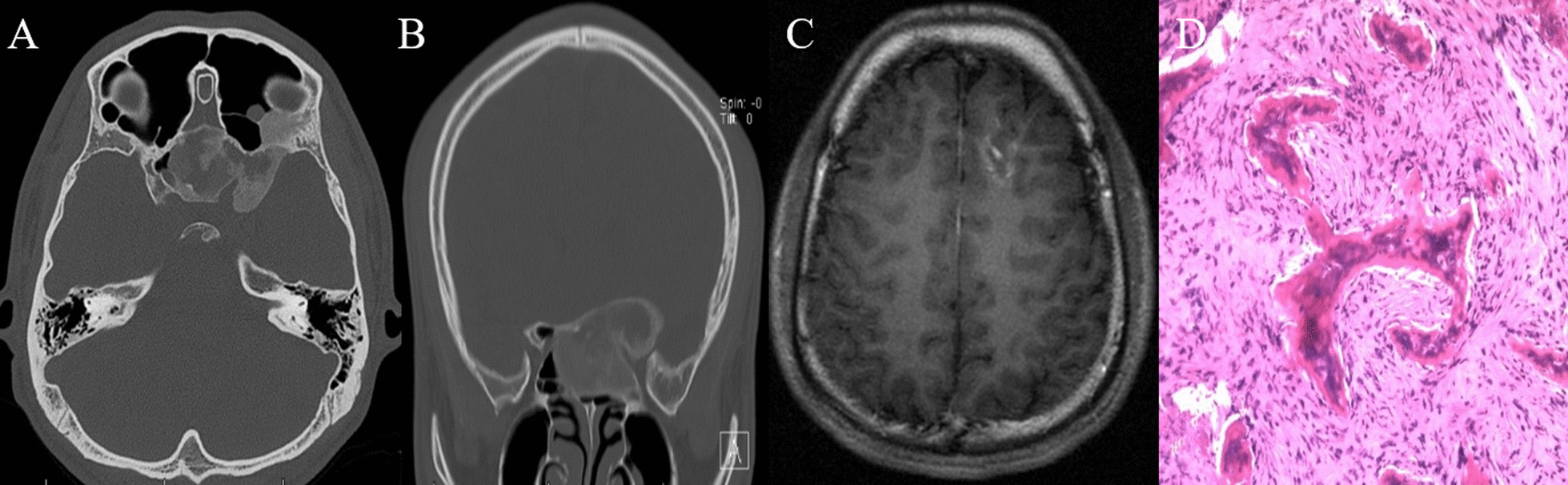
CT showing FD of sphenoid bone and left orbital apex (**A**, **B**). MRI showing venous malformation of the left frontal lobe (**C**). The patient’s FD was managed with transnasal surgery and the pathological report confirmed the FD diagnosis (**D**)

Patient was a 20-year-old female complaining about double vision, vision loss and exotropia of left eye. Her CT scans described abnormalities of her left sphenoid bone and temporal bone with typical radiological characteristics of FD (Fig. 2). At the same time, her MRI showed a venous malformation in left frontal lobe. The FD was resected through transnasal approach, and the pathological reports confirm the FD diagnosis. During the 2-year follow-up, his symptoms partially relieved and no recurrence was found radiologically.

#### Case 3

**Fig. 3:**
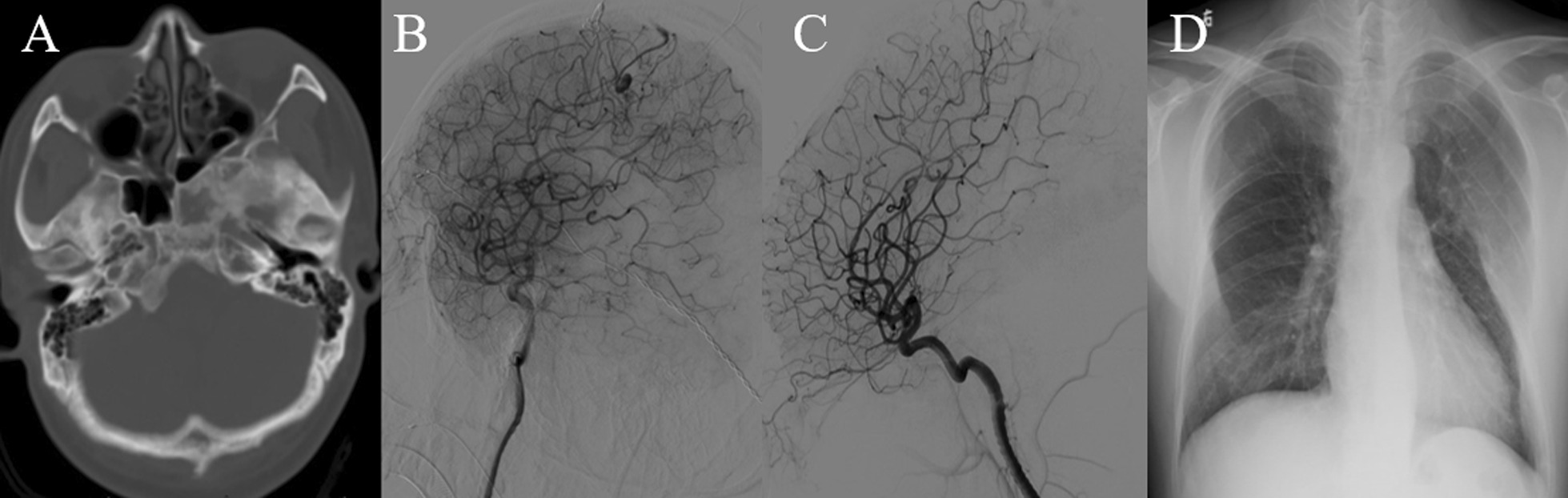
CT showing FD of the left sphenoid bone (**A**). DSA showing arteriovenous malformation in the right parietal lobe with right anterior cerebral artery being the major feeding artery and superior sagital sinus drainging (**B**). The lesion was resected completely (**C**). Chest X-ray showing FD of multiple ribs (**D**)

Patient was a 30 year-old male who was transferred to the emergency department for sudden headache, hemiplegia and loss of consciousness three years ago. His CT scan showed intracranial hemorrhage in the right parietal lobe and eternal ventricular drainage was done emergently (Fig. 3). After his symptoms relieved, the DSA found an AVM which was resected. For pre-operation evaluation, MRI and chest X-ray were done showing FD of the left sphenoid bone and bilateral ribs. One week after surgery, the patient was discharged home in good condition. After 1-year follow-up, the patient fully recovered and the radiological examinations showed no recurrence of AVM or progress of FD.

#### Case 4

**Fig. 4:**
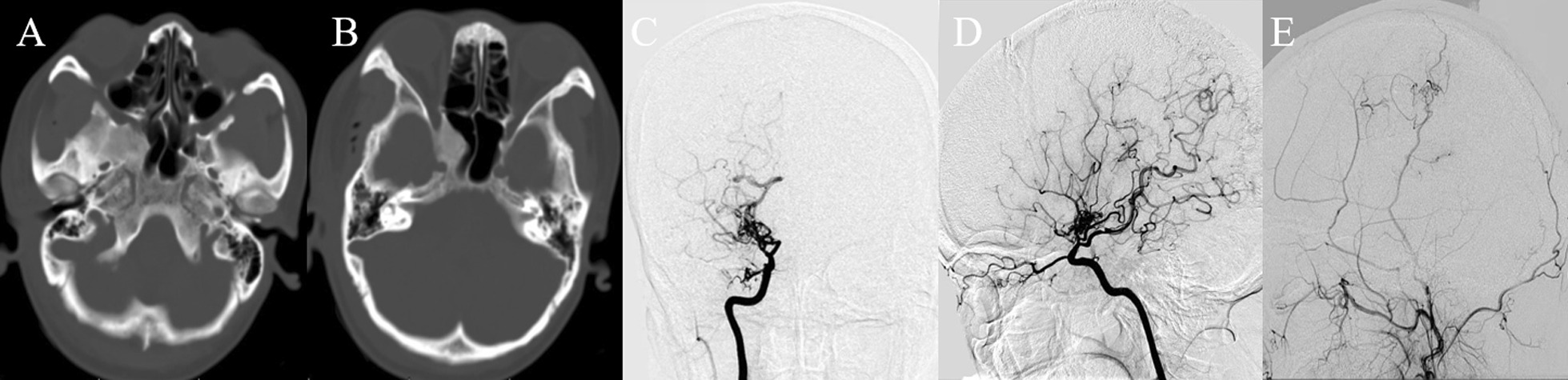
CT showing FD of local right sphenoid bone (**A**, **B**) and DSA showing MMD with the terminal of right internal carotid artery occluded (**C**, **D**). Right indirect revascularization was done and after 3 years, the DSA showed neovascularization (**E**)

Patient was a 34-year-old female suffering from recurrent transient ischemia attacks. Her radiological characteristics conformed with moyamoya disease (Fig. 4). Right encephalo-duro-arterio-synangiosis was performed successfully and her post-operation CT showed FD of the right sphenoid bone. Nine days after surgery, the patient was discharged home. During the 3-year follow-up, her symptoms got better and her radiological examinations showed neovascularization without progress of FD.

#### Case 5

**Fig. 5:**
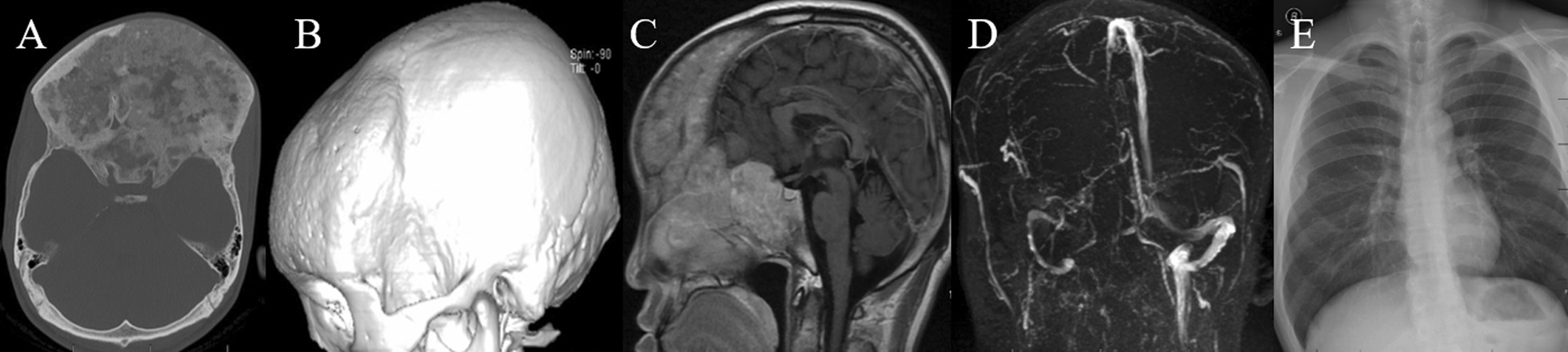
CT showing FD of Left ethmoid bone, nasal bone, sphenoid bone, bilateral orbits and frontal bones (**A**, **B**). MRI showing compression of the brain (**C**). MRV showing diffuse stenosis of the left venous sinuses (**D**). Chest X ray showing FD of the right 7th rib (E)

Patient was a 25-year-old male suffering from vision loss of left eye and weakness of right limbs. His CT scans showed extensive FD of left ethmoid bone, nasal bone, sphenoid bone, bilateral orbits and frontal bones with left brain tissue compressed and dislocated (Fig. 5). At the same time, the young patient was found to have FD of his 7th rib. MRA showed diffuse severe stenosis of venous sinuses of the left side. The patient was unwilling to receive any surgical treatment and was lost to follow-up.

#### Case 6

**Fig. 6:**
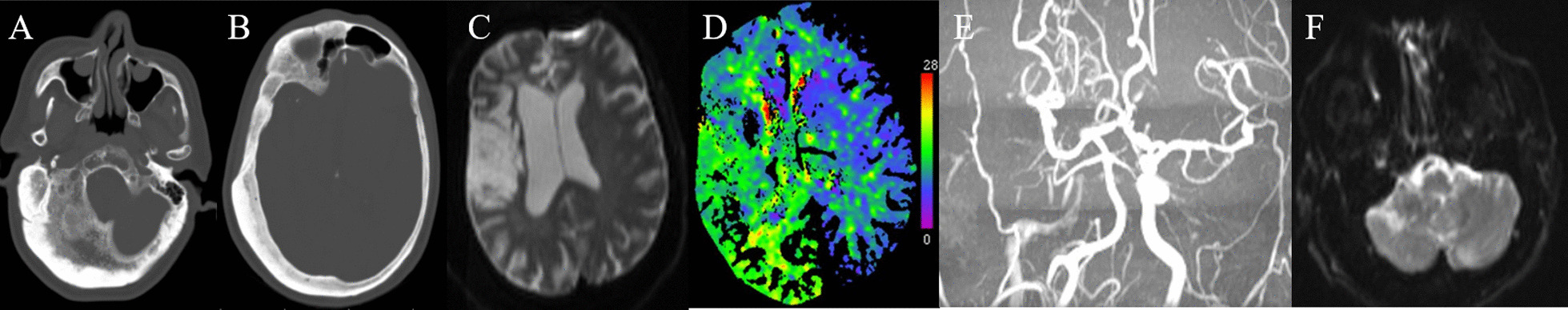
CT showing extensive FD of right skull bones especially the right skull base (**A**, **B**). MRI showing cerebral ischemia of right fronto-temporal lobes (**C**) and CTP showing diffuse hypoperfusion of the right brain hemisphere (**D**). MRA showing occlusion of the right internal carotid artery (**E**). During the 9-year follow-up, his ischemia progressed and MRI showed newly occurred cerebral ischemia of the right cerebellum (**F**)

Patient was a 44-year-old female admitted to the emergency department due to left facial paralysis and weakness of left limbs. DWI showed acute cerebral ischemia of right fronto-parietal lobe (Fig. 6). MRA showed complete occlusion of the right internal artery and CT showed extensive craniofacial FD involving the right frontal bone, temporal bone, parietal bone and the occipital bone especially the right skull base. The male patient was managed conservatively with medical treatment with close follow-up for 9 years during which time he suffered from recurrent acute cerebral ischemia and MRI showed progress of brain ischemia involving right fronto-parietal lobe and right cerebellum.

#### Case 7

**Fig. 7:**
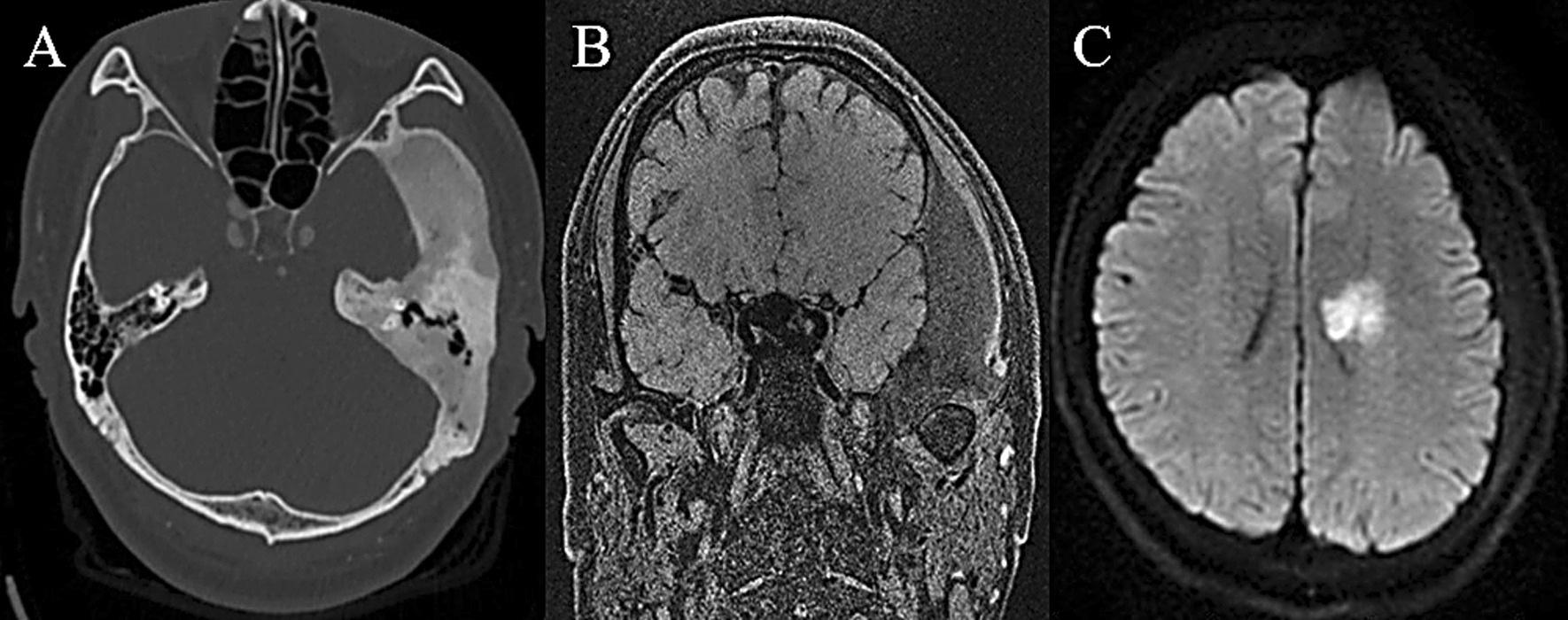
CT showing FD of the left parietal bone and temporal bone (**A**). MRI showing compression of the brain tissue (**B**) and acute cerebral ischemia of the left basal ganglia (**C**)

Patient was a 36-year-old male with chief complaint being aphasia and right facial paralysis. MRI showed acute cerebral ischemia of left basal ganglia (Fig. 7) and the patient was treated medically. However, the patient’s symptoms remained the same. In addition, the ultrasound showed diffuse thyroid disease and multiple thyroid nodules. After 12 days in hospital, the patient was discharged home but was lost to follow-up.

## Discussion

As we know, this article was the first report worldwide discussing the coexistence of the CFD and CVD. There might be three possible explanations for the coexistence: (1) genetic predisposition which means they may share something in common etiologically and pathophysiologically; (2) environmental influence that is to say one disease might have influence on the development, management and prognosis of the other; (3) chance occurrence thus having no mutual interaction.

CFD has been reported to be coexisted with some central nervous system diseases such as meningioma [11–13]*,* plasmacytoma [14], Chondrosarcoma [15] and pituitary adenomas [16]. What’s more, some literatures deduced that it was highly likely to cause neurovascular impingement [17]. Early in the twentieth century, Joseph et al. described the angiographic features of fibrous dysplasia of the skull in two patients including the local displacement of the vessels, abnormal vessel dilation, abnormal small beaded vessels, and arterial and venous aneurysm formations [18]. Several case reports published describing FD coexisting with craniofacial vascular malformations such as occipital intraosseous AVF [7] and AVM involving the palpebra and orbit [4].

Our series covered almost all the clinical types of cerebrovascular diseases which is the first to focus on the relationship of CFD and vascular diseases. In our review of the previous case reports of this coexistence, which is pretty rare, all of the coexisted vascular diseases described were intraosseous AVF/AVM. And our series is fairly different.

Currently there are no known epidemiological or well-characterized genetic associations between CFD and CVD. FD arises from post-zygotic gain-of-function somatic mutations in the GNAS gene [19]. Multiple tissues may be affected leading to multiple clinical abnormalities including neurological abnormalities which are influenced by the time and developmental stage when these kind of mutations occur and by which parental allele becomes mutated [20, 21]. It has been proven that there is various heritable contributions to the pathogenesis of CVD. For instance, the formation of IAs are related with significant genetic heterogeneity especially CDKN2A-CDKN2BAS, ADAMTS, ELN, SOX17, HDAC9, EDNRA and so on [22–30]. And recurrently mutated genes were identified in several vascular malformations as well including TEK/TIE2 mainly in venous malformations [31], GNA11/14 mainly in vascular tumors [32], KRAS/NRAS/RASA1/HRAS mainly in AVMs [33], GNAQ [34], IDH1/2 [35], AKT1, PTEN and PIK3CA [36]. What’s more, moyamoya diseases, which used to be considered as a rare polygenic cerebrovascular disease, is also known to be associated with some susceptibility genes especially RNF213 p.R4810K and human leukocyte antigen (HLA) [37]. Additionally, there is also a significant association of the RNF213 variant with ischemic stroke especially with cervical/intracranial large artery atherosclerosis [38]. These mutations are believed to be closely related with angiogenesis, inflammatory response [39], cell growth, proliferation, survival, and cell cycle progression [36]. As above, in respect of etiology, although the occurrence of FD and CVD such as IAs, cerebral vascular malformations and moyamoya disease are supported to have something to do with the early gene mutations during embryo genesis [40], they may not share the same variant or the common pathophysiological pathway/mechanism.

When it comes to disease development, although no exact gene mutations is known to be shared by the two kinds of diseases, the fact that they both have some abnormal embryological development might imply that there may be some common but yet undetected influencing factors between the two diseases.

Firstly, the common dilated vascular channels and foci of interstitial and peritrabecular hemorrhage in FD may probably explain the propensity to develop vascular anomalies [41] such as AVFs [9, 42, 43] and AVMs [5, 8]. Secondly, another possible explanation for the coexistence of FD and hemorrhagic cerebrovascular diseases probably lies in the hemodynamics. Most of the CFD involves the skull base especially the sphenoid bone where the circle of willis which is the predilection site of IAs is located on. FD of this site might result in the displacement of the artery thus altering the angle of arterial bifurcations and subsequently contributing to the formation of IAs to some degree. However, since there was no other study focusing on the relationship between craniofacial FD and vascular diseases and consider the lack of hemodynamic evaluations in our case series, we cannot provide solid evidence for our hypothesis.

It is known that FD in the skull base especially in the sphenoid and temporal bones may result in vision and hearing loss due to the constriction of the cranial nerves caused by the hyperostosis of the involved bones, making us wondering whether this disease could have similar impact on the cerebral vessels leading to not only displacement but also encircling of the vessels leading to stenosis even occlusion of the affected vessels. Additionally, the thickening of the bones attributed to craniofacial FD might also result in the narrowing of the cranial capacity resulting in the increase of intracranial pressure and hemodynamic changes, which is thought to be associated with cerebrovascular stenosis/occlusion and the decrease of cerebral perfusion. The encircling of intracranial arteries is probably consistent with the case 6 in our series. The 44-year old male patient suffered from recurrent cerebral ischemic stroke for the occlusion of right internal carotid artery. The reason why the occlusion occurred in a relative young age may be attributed to the extensive FD of the right skull base which may cause constriction of the right internal carotid artery especially in the area of carotid canal through which the internal carotid artery goes into the cranial cavity. And the increase of intracranial pressure resulted from the narrowing of the cranial capacity might aggravate the ischemic situation. In case 5, the 25-year old boy suffered from extensive stenosis of his left intracranial venous sinus, which might caused by increase of the intracranial pressure. And in case 7, the patient was diagnosed with acute cerebral ischemia without any abnormalies of his cervical and intracranial arteries which might be explained by the hypoperfusion caused by the increase of intracranial pressure.

With respect to the management, on the one hand, the coexistence of craniofacial FD may cause difficulties during surgery for the cerebrovascular diseases such as tedious bone work due to extremely thickened bones or massive blood loss due to the hypervascular bones. What’s more because of the thickened bone, larger exposure was needed and more angulation of microscope was required to reach the lesion along with increased retraction of ipsilateral cerebellar lobes. Therefore, clinicians need to adopt individualized management for this coexistence. In our series, both craniotomy and endovascular management were adopted unlike in the literature review, only endovascular treatment was done to manage the coexisted vascular diseases. On the other hand, the coexistence of CFD and vascular diseases indicate that clinicians need to fully aware of possible alterations of hemodynamics and intracranial pressure caused by CFD and the resulted CVD which was totally neglected before, and the multidisciplinary team might be needed for the evaluation and management of the disease. It is reasonable and highly recommended for patients with CFD to do throughout examinations evaluating not only the disease burden of FD since it tend to be polyosotic and have multiple clinical manifestations but also the intracranial vessels which might allow the physicians to detect the coexisting vascular diseases on time thus individualizing the treatment strategies, reduce the treatment risks and improve patients’ diagnosis.

## Limitation

Firstly, due to the retrospective nature of the article, we did not have access to throughout patient information therefore we could not provide definite explanation for the rare coexistence. Secondly, due to the rareness of CFD, the sample size was fairly small making it unable for us to do further statistical analysis for more evidence and only simple description of the series could be done.

## Conclusion

The coexisting of these lesions in central nervous system reported could not only provide clinicians with further knowledge of FD but also could help them understand the etiology of these lesions. And considering about the possible interaction between these two diseases, it is fair reasonable for patients with CFD to have some screenings and evaluations about CVDs.

## Data Availability

The datasets generated and/or analysed during the current study are not publicly available due to individual privacy of the patients included but are available from the corresponding author on reasonable request.
